# Relationship between sexually transmitted infections knowledge and the sexual behavior of Brazilian future doctors

**DOI:** 10.3389/fmed.2024.1512590

**Published:** 2024-12-24

**Authors:** Maria Clara Sales do Nascimento, Luiz Ricardo Cerqueira Freitas Junior, Isabel Carmen Fonseca Freitas, Katia de Miranda Avena, Bruno Bezerril Andrade

**Affiliations:** ^1^Medical College, Bahiana School of Medicine and Public Health, Salvador, Brazil; ^2^Medical College, Bahia State University, Salvador, Brazil; ^3^Department of Pediatrics, School of Medicine, Federal University of Bahia, Salvador, Brazil; ^4^Zarns Medical College, Clariens Educação, Salvador, Bahia, Brazil; ^5^Laboratory of Clincial and Translational Research, Gonçalo Moniz Institute, Oswaldo Cruz Foundation, Salvador, Bahia, Brazil

**Keywords:** sexually transmitted diseases, sexual behavior, medical students, sex education, health risk behaviors

## Abstract

**Background:**

The prevalence of sexually transmitted infections (STIs) remains alarming, especially among young people and college students, highlighting the vulnerability of this population. In the academic context, it is worth investigating whether medical students, despite their access to information, also engage in risky sexual behaviors.

**Objective:**

The present study aims to describe the sexual behavior of Brazilian medical students, analyzing their level of knowledge about HIV/AIDS and other STIs, as well as examining potential correlations between this knowledge and risky behaviors.

**Methods:**

This cross-sectional study was conducted with 193 medical students from a private institution in Salvador, Bahia, Brazil, using a structured, anonymous, self-administered online questionnaire. The questionnaire was adapted from the Brazilian Knowledge, Attitudes, and Practices Survey and a survey on risk behavior and knowledge among university students. Knowledge about STIs and HIV/AIDS was classified as “good” (above 70% correct answers), “average” (50–70% correct answers), and “poor” (below 50% correct answers). The work has been approved by the institutional review board of Bahiana School of Medicine and Public Health.

**Results:**

A total of 77.7% of the students exhibited risky sexual behavior, which was associated with the number of partners (*p* = 0.0001), engaging in sexual activity within the last 12 months (*p* = 0.001), lack of a steady partner (*p* = 0.001), not using condoms during the last sexual encounter with a steady partner (*p* = 0.0001), and the use of substances such as alcohol (*p* = 0.0001), marijuana (*p* = 0.0001), and cigarettes (*p* = 0.0001) during sexual activity. Most students demonstrated poor (49.2%) or average (48.7%) knowledge about STIs and HIV/AIDS, a pattern that persisted regardless of sexual behavior. Only not using condoms during sexual encounters with casual partners in the past 12 months (*p* = 0.021) was associated with low levels of knowledge.

**Conclusion:**

The prevalence of risky sexual behaviors in this sample was high, as was the low level of knowledge about STIs. However, knowledge of STIs and HIV/AIDS does not appear to be the sole determinant of these behaviors.

## 1 Introduction

Sexually transmitted infections (STIs) represent a persistent and substantial public health challenge, affecting millions of people both in Brazil and globally (1). Estimates indicate that approximately 360 million cases of STIs occur worldwide each year, with 10 to 12 million cases registered in Brazil (2). Previous studies have shown that the burden of STIs is disproportionately high among adolescents and young adults, with higher rates observed in specific regions and populations (3, 4), underlining the importance of preventive strategies tailored to these groups. These data underscore the need for effective strategies for the prevention and control of these diseases, especially among young people and college students, who are among the most vulnerable groups (5).

Risky sexual behaviors, such as engaging in unprotected sex, having multiple partners, and drug use, play a fundamental role in the spread of STIs, particularly among adolescents and young adults aged 15 to 24 (6). These behaviors are influenced by a complex interaction of social, cultural, psychological, and biological factors that shape the maturation process and are closely related to the university environment (7). Studies have shown that the university environment often contributes to a sense of anonymity and freedom, which can lead to risky behaviors such as multiple sexual partners (8) and alcohol use (8–10). Furthermore, peer pressure and the desire for social acceptance are commonly cited as significant factors influencing these behaviors among young adults (11).

The university academic setting, characterized by greater individual freedom and changes in social interactions, often creates an atmosphere conducive to the emergence and consolidation of risky behaviors, such as alcohol consumption (9) and unprotected sex (5, 7). This vulnerability is exacerbated by inadequate preparation to deal with sexuality-related issues and insufficient knowledge of the topic (2, 6, 12). Investigations into medical students’ knowledge of sexual health revealed significant gaps in understanding about STIs transmission and prevention (13, 14). Despite being exposed to comprehensive medical curricula, many students report feeling unprepared to address sexual health topics effectively in clinical practice (15, 16).

In addition to individual vulnerability, it is also important to consider that health students bear an even greater responsibility, as they may be tasked with guiding the public on sexual and reproductive health during their training. This underscores the need for a consistent and early understanding of these issues. However, despite having access to extensive educational resources, medical students often struggle to translate their knowledge into safer behaviors (17–19). Previous studies have highlighted the discrepancy between medical students’ knowledge of sexual health and their actual clinical practices (14, 20). This gap between theoretical knowledge and practical behavior becomes a critical point of investigation, particularly for future doctors, who will be responsible for disseminating knowledge and promoting preventive practices.

Given the relevance of this topic and the pressing need to understand and address the factors contributing to the risky sexual behaviors of these future doctors, the present study aimed to describe the sexual behavior of medical students, analyze their sociodemographic profile, and assess their level of knowledge about HIV/AIDS and other STIs. The study also sought to identify potential correlations between knowledge and risky behaviors, with the goal of contributing to the development of more targeted and effective preventive interventions for this audience.

## 2 Methods

### 2.1 Study design

A cross-sectional study was conducted from March 25 to May 25, 2021, in the city of Salvador, Bahia, Brazil.

### 2.2 Population and sample

The target population consisted of medical students from a private higher education institution in Brazil.

The study sample included students from the first, third, and sixth years of the medical course, aged over 18, who were officially enrolled. This selection was intentional, aiming to evaluate the progression of knowledge about risky sexual behavior across the three academic cycles: basic (1st to 4th semesters), clinical (5th to 8th semesters), and internship (9th to 12th semesters). Students who reported not having had sexual relations at the time of the survey or provided incomplete responses to the questionnaire were excluded from the analysis.

The sample size was estimated considering a target population of 900 students enrolled at the institution. To ensure a 95% confidence level and a 5% margin of error, the sample size was estimated at 194 students, ensuring the statistical robustness needed for data analysis. Ultimately, 213 responses were obtained, representing a response rate of 23.7%, surpassing the initially estimated sample size and reinforcing the statistical validity of the results.

### 2.3 Procedures and measurements

Data collection was conducted through a structured, anonymous, self-administered virtual questionnaire created using Google Forms. All enrolled students were invited to participate via an email invitation containing the link to access the questionnaire. The invitation was sent to the students’ institutional email, which is generated by the university upon enrollment. At this institution, each academic semester is associated with a specific institutional email list, which includes the email addresses of all students enrolled in that semester. As a result, an informational email with the questionnaire link was sent to each of these addresses, ensuring that the entire student body had access to the invitation to participate. Before accessing the questionnaire, students were required to provide formal consent via the Informed Consent Form (ICF) available on the form’s homepage.

Data collection was based on a questionnaire adapted from two previous studies: the Knowledge, Attitudes, and Practices Survey of the Brazilian population (PCAP) (21) and the Survey on Risk Behavior and Knowledge in University Students (19). The PCAP is a survey validated by the Brazilian Ministry of Health and is widely used to assess behavior and knowledge regarding STIs in Brazil (21). Since it has five independent sections that can be analyzed separately, it does not require a validation process for its adaptation (21). In this study, only sections 1 (socioeconomic conditions), 2 (knowledge of HIV/AIDS and other STI transmission), and 5 (drug use and sexual practices) were used.

The questionnaire comprised 39 objective questions, covering sociodemographic aspects (questions 1 to 8), STI transmission methods (questions 9 to 18), sexual behavior (questions 19 to 23), sexual experiences in the past 12 months (questions 24 to 34), and the use of licit and illicit drugs during sexual relations (questions 35 to 39).

To assess social, cultural, and economic aspects, data were collected on sex, age, marital status, religion, family income, and the educational level of both the family and the student.

Regarding the evaluation of sexual behavior, risky behavior was defined as the lifetime practice of two or more of the following sexual behaviors: (I) inconsistent condom use; (II) more than 10 sexual partners; (III) sexual intercourse after alcohol consumption; (IV) sexual intercourse after illicit substance use; and (V) sexual intercourse with a person little known or recently acquainted (22).

Knowledge about STIs and HIV/AIDS was assessed based on responses to questions 9 to 18. For each question, answers were classified as correct or incorrect according to a predefined standard. The option “I do not know” was treated as an incorrect response. Additionally, the level of knowledge was categorized into three levels: “good” (above 70% correct), “average” (50–70% correct), and “poor” (below 50% correct) (23).

### 2.4 Statistical analysis

Data were tabulated in Microsoft Excel and analyzed using IBM SPSS Statistics, version 25.0.

The data analysis employed both descriptive and inferential statistics. Continuous variables were expressed as means and standard deviations (M ± SD), and categorical variables as absolute and relative frequencies.

To verify the association between (I) knowledge level and student profile, (II) knowledge level and sexual behavior, and (III) sexual behavior and student profile, the chi-square test was applied. When the expected count in one or more cells of the contingency table was less than five, Fisher’s exact test was used. Associations were considered statistically significant at *p* < 0.05.

Additionally, Cramér’s V coefficient was calculated to assess the strength of the observed associations. Cramér’s V was interpreted according to conventional values: <0.1 indicated a weak association, between 0.1 and 0.3 indicated a moderate association, and > 0.3 indicated a strong association.

### 2.5 Ethical considerations

This study was conducted in accordance with Resolutions 466/12 and 510/16 of the National Health Council. The project was approved by the Research Ethics Committee of the Bahiana School of Medicine and Public Health (protocol no. 40394920.3.0000.5544, report no. 4.612.617). The autonomy, confidentiality, and privacy of the participants were carefully maintained throughout the study. Data confidentiality and participant privacy were ensured by restricting access to information exclusively to the researchers involved. All study participants were fully informed about the research objectives and methods and provided their consent by digitally signing the ICF.

## 3 Results

Out of the 213 responses, 20 were excluded as they came from students who had not yet initiated sexual activity, leaving a final sample of 193 medical students. No incomplete questionnaires were identified. Among these, there was a predominance of third-year medical students (69.4%), with a mean age of 22.3 ± 3.4 years. The majority were female (70.5%), single (92.2%), and non-religious (56.5%); among those who practiced a religion, Catholicism was the most prevalent (57.1%). Most students had no prior academic training (87.0%) and belonged to families with an income exceeding 10 minimum wages (58.0%; Table 1).

**Table 1:** Sociodemographic characteristics of study participants (*n* = 193).

Characteristics	MA ± SD	n (%)
Age		
In years	22.3 ± 3.4	
Sex		
Male		57 (29.5)
Female		136 (70.5)
Marital status		
Single		178 (92.2)
Married		12 (6.2)
Separated/Divorced		3 (1.6)
Practicing a religion		
No		109 (56.5)
Yes		84 (43.5)
Religion practiced*		
Catholic		48 (57.1)
Evangelical		17 (20.2)
Spiritist		17 (20.2)
African-based religions		2 (2.5)
Academic year		
1st Year		45 (23.3)
3rd Year		134 (69.4)
6th Year		14 (7.3)
Previous academic background		
No		168 (87.0)
Yes		25 (13.0)
Family income**		
1 to 4 minimum wages		36 (18.7)
5 to 9 minimum wages		45 (23.3)
>10 minimum wages		112 (58.0)

MA, mean arithmetic; SD, standard deviation; n, frequency of categories in absolute number; %, frequency of categories in percentage; *Relative frequency calculated considering a sample of 84 religiously active students; **The minimum wage in Brazil is approximately $291.12.

### 3.1 Sexual behavior of students

Among medical students who had initiated their sexual activity, 77.7% reported risky sexual behaviors. The first sexual intercourse occurred, on average, at 17.3 ± 2.4 years, with 72.0% reporting condom use at that time. Furthermore, 79.3% stated that they had had fewer than 10 sexual partners up to that point, and 69.8% used condoms during their last sexual encounter. However, 67.4% of the students had not engaged in sexual activity in the past 12 months. Among those who maintained a steady partner during this period (64.8%), 63.2% did not use condoms during their last encounter, and among those who did use condoms, 50.0% did not use them consistently. Among students without a steady partner, 69.8% used condoms in casual encounters in the past 12 months, but only 38.6% used them consistently. Additionally, 29.0% of students reported having sexual relations with individuals met online, and 32.6% engaged in sexual activity under the influence of substances such as alcohol (70.5%), marijuana (23.8%), cigarettes (18.7%), crack (1.0%), or cocaine (1.0%; Table 2).

**Table 2:** Sexual behavior of study participants (*n* = 193).

Behavior	MA ± SD	n (%)
Risky sexual behavior, n (%)		
No		43 (22.3)
Yes		150 (77.7)
Age at first sexual relation		
Years, MA ± SD	17.3 ± 2.4	
Sexual partners > 10, n (%)		
No		153 (79.3)
Yes		40 (20.7)
Sexual relation in the last 12 months, n (%)		
No		130 (67.4)
Yes		63 (32.6)
Fixed partner in the last 12 months, n (%)		
No		68 (35.2)
Yes		125 (64.8)
Condom use		
At first sexual relation, n (%)		
No		54 (28.0)
Yes		139 (72.0)
At last sexual relation*, n (%)		
No		19 (30.2)
Yes		44 (69.8)
At last relation with fixed partner in the last 12 months**, n (%)		
No		79 (63.2)
Yes		46 (36.8)
In all sexual relations with fixed partner***, n (%)		
No		23 (50.0)
Yes		23 (50.0)
In relation with hookups/casual encounters in the last 12 months****, n (%)		
No		19 (30.2)
Yes		44 (69.8)
In all sexual relations with hookups/casual encounters*****, n (%)		
No		27 (61.4)
Yes		17 (38.6)
Sexual relation		
With known individuals on the internet, n (%)		
No		137 (71.0)
Yes		56 (29.0)
With hookups/casual encounters in the last 12 months, n (%)		
No		130 (67.4)
Yes		63 (32.6)
Under the influence of alcohol, n (%)		
No		57 (29.5)
Yes		136 (70.5)
Under the influence of Marijuana, n (%)		
No		147 (76.2)
Yes		46 (23.8)
Under the influence of crack, n (%)		
No		191 (99.0)
Yes		2 (1.0)
Under the influence of cigarettes, n (%)		
No		157 (81.3)
Yes		36 (18.7)
Under the influence of cocaine, n (%)		
No		191 (99.0)
Yes		2 (1.0)

MA, mean arithmetic; SD, standard deviation; n, frequency of categories in absolute number; %, frequency of categories in percentage; *Refers to students who engaged in sexual activity in the past 12 months; **Relative frequency calculated considering a sample of 125 students with a steady partner; ***Relative frequency calculated considering a sample of 46 students who used condoms during their last encounter with a steady partner in the past 12 months; ****Relative frequency calculated considering a sample of 68 students without a steady partner; ***** Relative frequency calculated considering a sample of 44 students who used condoms in encounters with casual partners in the past 12 months.

### 3.2 Association between sexual behavior and student profile

Significant associations were found between risky sexual behaviors and various characteristics of the medical students’ profile. A strong correlation was noted between the number of sexual partners (*p* = 0.0001, Cramer’s V = 0.265), engaging in sexual activity in the past 12 months (*p* = 0.001, Cramer’s V = 0.519), and the absence of a steady partner (*p* = 0.001, Cramer’s V = 0.519), with an increase in other risky behaviors. Moreover, condom use proved to be a relevant factor, with a significant association between the absence of condom use during the last sexual encounter with a steady partner and other risky sexual behaviors (*p* = 0.0001, Cramer’s V = 0.234). Statistically relevant correlations were also identified between risky sexual behavior and the use of substances such as alcohol (*p* = 0.0001, Cramer’s V = 0.363), marijuana (*p* = 0.0001, Cramer’s V = 0.300), and cigarettes (*p* = 0.0001, Cramer’s V = 0.256) during sexual encounters. Other factors, such as gender, marital status, family income, academic profile, and religious practice, did not present significant associations (Table 3).

**Table 3:** Association between sexual behavior and the sociodemographic profile of study participants (*n* = 193).

Variables, n (%)	Risky sexual behavior		p*	*Cramer’s V^§^*
	No	Yes		
Sex			0.574	0.046
Male	11 (19.3)	46 (80.7)		
Female	32 (23.5)	104 (76.5)		
Marital status			0.303	0.111
Single	42 (23.6)	136 (76.4)		
Married	0 (0.0)	3 (100.0)		
Separated/Divorced	1 (8.3)	11 (91.7)		
Practicing a religion			0.863	0.018
No	25 (22.9)	84 (77.1)		
Yes	18 (21.4)	66 (78.6)		
Academic year			0.062	0.170
1st Year	6 (13.3)	39 (86.7)		
3rd Year	36 (26.9)	98 (73.1)		
6th Year	1 (7.1)	13 (92.9)		
Previous academic background			0.607	0.058
No	39 (23.2)	129 (76.8)		
Yes	4 (16.0)	21 (84.0)		
Family income^€^			0.919	0.030
1 to 4 minimum wages	8 (22.2)	28 (77.8)		
5 to 9 minimum wages	11 (24.4)	34 (75.6)		
>10 minimum wages	24 (21.4)	88 (78.6)		
Sexual partners > 10				0.265
No	43 (27.7)	112 (72.3)		
Yes	0 (0.0)	38 (100.0)		
Sexual relation in the last 12 months				0.459
No	22 (62.9)	13 (37.1)		
Yes	21 (13.3)	137 (86.7)		
Fixed partner in the last 12 months				0.234
No	24 (35.3)	44 (64.7)		
Yes	19 (15.2)	106 (84.8)		
Condom use				
At first sexual relation			0.084	0.112
No	8 (14.8)	46 (85.2)		
Yes	35 (25.2)	104 (74.8)		
At last sexual relation*			0.081	0.114
No	23 (18.7)	100 (81.3)		
Yes	20 (28.6)	50 (71.4)		
At last relation with fixed partner in the last 12 months**				0.519
No	0 (0.0)	75 (100.0)		
Yes	19 (38.0)	31 (62.0)		
In all sexual relations with fixed partner***			0.144	0.275
No	0 (0.0)	27 (100.0)		
Yes	2 (11.8)	15 (88.2)		
In relation with hookups/casual encounters in the last 12 months****			0.484	0.119
No	0 (0.0)	19 (100.0)		
Yes	2 (4.5)	42 (95.5)		
In all sexual relations with hookups/casual encounters*****			0.144	0.275
No	0 (0.0)	27 (100.0)		
Yes	2 (11.8)	15 (88.2)		
Sexual relation				
With known individuals on the internet				0.287
No	2 (3.6)	54 (96.4)		
Yes	41 (29.9)	96 (70.1)		
With hookups/casual encounters in the last 12 months				0.329
No	2 (3.1)	63 (96.9)		
Yes	41 (32.0)	87 (68.0)		
Under the influence of alcohol				0.363
No	26 (45.6)	31 (54.4)		
Yes	17 (12.5)	119 (87.5)		
Under the influence of marijuana				0.300
No	43 (29.3)	104 (70.7)		
Yes	0 (0.0)	46 (100.0)		
Under the influence of crack			0.603	0.055
No	41 (32.0)	87 (68.0)		
Yes	0 (0.0)	2 (100.0)		
Under the influence of cigarettes				0.256
No	43 (27.4)	114 (72.6)		
Yes	0 (0.0)	36 (100.0)		
Under the influence of cocaine			0.603	0.055
No	43 (22.5)	148 (77.5)		
Yes	0 (0.0)	2 (100.0)		

n, frequency of categories in absolute number; %, frequency of categories in percentage; ^€^The minimum wage in Brazil is approximately $291.12; *Chi-square test or Fisher’s test; ^§^Cramer’s Correlation Coefficient; **Relative frequency calculated considering a sample of 125 students with a steady partner; ***Relative frequency calculated considering a sample of 46 students who used condoms during their last encounter with a steady partner in the past 12 months; ****Relative frequency calculated considering a sample of 68 students without a steady partner; ***** Relative frequency calculated considering a sample of 44 students who used condoms in encounters with casual partners in the past 12 months.

### 3.3 Level of knowledge of students about STIs and HIV/AIDS

When analyzing the level of knowledge about STIs and HIV/AIDS among all participants in the study, it was observed that the majority of students had knowledge classified as poor (49.2%) or fair (48.7%). This pattern remained consistent regardless of sexual behavior, with most students who engaged in risky behaviors classified as having poor (47.3%) or fair (50.7%) knowledge; similarly, those who did not exhibit risky sexual behaviors were also predominantly classified as having poor (55.8%) or fair (41.9%) knowledge, suggesting that while the level of knowledge is important, it did not appear to be the sole determining factor in the adoption of risky behaviors (Table 4).

**Table 4:** Knowledge level of study participants about STIs and HIV/AIDS according to their sexual behavior (*n* = 193).

Risky sexual behavior	Level of knowledge*			Total
	Poor	Average	Good	
Yes	71 (47.3)	76 (50.7)	3 (2.0)	150 (77.7)
No	24 (55.8)	18 (41.9)	1 (2.3)	43 (22.3)
Total	95 (49.2)	94 (48.7)	4 (2.1)	193 (100.0)

*Levels of knowledge: “good”—above 70% correct; “average”—50–70% correct; and “poor”—below 50% correct.

The analysis of the content addressed revealed that questions related to HIV/AIDS transmission, such as the use of condoms to prevent transmission and the fact that seemingly healthy individuals may be infected, had high correct response rates (96.9 and 98.4%, respectively). However, misconceptions persist, such as the transmission of diseases through not using condoms during sexual relations or sharing syringes or needles with others (99.9 and 97.9% incorrect responses, respectively) and regarding diseases that have a cure (83.4% incorrect responses), indicating significant knowledge gaps (Table 5).

**Table 5:** Evaluation of knowledge about STIs and HIV/AIDS among study participants (*n* = 193).

Questions	Answers	
	Correct	Incorrect
Which of the diseases described in the list can a person be infected with after being bitten by an insect, such as a mosquito?	163 (84.5)	30 (15.5)
Can the risk of HIV/AIDS transmission be reduced if a person only has sexual relations with a faithful and uninfected partner?	154 (79.8)	39 (20.2)
Can a healthy-looking person be infected with the HIV/AIDS virus?	190 (98.4)	3 (1.6)
Is using a condom the best way to prevent the HIV/AIDS virus from being transmitted during sexual intercourse?	187 (96.9)	6 (3.1)
Can a person be infected with the HIV/AIDS virus by sharing utensils, glasses, or meals?	172 (89.1)	21 (10.9)
Can a pregnant woman with the HIV/AIDS virus, who receives specific treatment during pregnancy and delivery, reduce the risk of transmitting the virus to her child?	168 (87.0)	25 (13.0)
Which of the diseases described in the list can a person be infected with by using public restrooms?	76 (39.3)	117 (60.7)
Which of the diseases described in the list can a person be infected with by sharing needles or syringes with others?	4 (2.1)	189 (97.9)
Which of the diseases described in the list can a person be infected with by not using a condom during sexual intercourse?	2 (1.0)	191 (99.0)
For which of the diseases described in the list is there a cure?	32 (16.6)	161 (83.4)

### 3.4 Association between level of knowledge about STIs and HIV/AIDS and student profile

The analysis of the association between the level of knowledge about STIs and HIV/AIDS and the profile of medical students revealed no significant associations in most of the evaluated variables, except for the non-use of condoms in encounters with casual partners in the past 12 months (*p* = 0.021, Cramer’s V = 0.350). Individuals with lower knowledge levels used condoms less frequently (Table 6).

**Table 6:** Association between the level of knowledge about STIs and HIV/AIDS and the profile of study participants (*n* = 193).

Variables, n (%)	Level of knowledge^£^			p*	Cramer’s V^§^
	Poor	Average	Good		
Sex				0.743	0.024
Male	29 (50.9)	27 (47.4)	1 (1.8)		
Female	66 (48.5)	67 (49.3)	3 (2.2)		
Marital status				0.165	0.130
Single	83 (46.6)	91 (51.1)	4 (2.2)		
Married	2 (66.7)	1 (33.3)	0 (0.0)		
Separated/Divorced	10 (83.3)	2 (16.7)	0 (0.0)		
Practicing a religion				0.071	0.166
No	55 (50.5)	54 (49.5)	0 (0.0)		
Yes	40 (47.6)	40 (47.6)	4 (4.8)		
Academic year				0.235	0.120
1st Year	25 (55.6)	18 (40.0)	2 (4.4)		
3rd Year	64 (47.8)	69 (51.5)	1 (0.7)		
6th Year	6 (42.9)	7 (50.0)	1 (7.1)		
Previous academic background				0.398	0.098
No	85 (50.6)	79 (47.0)	4 (2.4)		
Yes	10 (40.0)	15 (60.0)	0 (0.0)		
Family income^€^				0.341	0.108
1 to 4 minimum wages	16 (44.4)	18 (50.0)	2 (5.6)		
5 to 9 minimum wages	26 (57.8)	19 (42.2)	0 (0.0)		
>10 minimum wages	53 (47.3)	57 (50.9)	2 (1.8)		
Sexual partners > 10				0.845	0.042
No	75 (48.4)	77 (49.7)	3 (1.9)		
Yes	20 (52.6)	17 (44.7)	1 (2.6)		
Sexual relation in the last 12 months				0.168	0.136
No	22 (62.9)	12 (34.3)	1 (2.9)		
Yes	73 (46.2)	82 (51.9)	3 (1.9)		
Fixed partner in the last 12 months				0.242	0.121
No	33 (48.5)	32 (47.1)	3 (4.4)		
Yes	62 (49.6)	62 (49.6)	1 (0.8)		
Condom use					
At first sexual relation				0.062	0.170
No	21 (38.9)	33 (61.1)	0 (0.0)		
Yes	74 (53.2)	61 (43.9)	4 (2.9)		
At last sexual relation*				0.328	0.108
No	65 (52.8)	55 (44.7)	3 (2.4)		
Yes	30 (42.9)	39 (55.7)	1 (1.4)		
At last relation with fixed partner in the last 12 months**				0.715	0.073
No	37 (49.3)	37 (49.3)	1 (1.3)		
Yes	25 (50.0)	25 (50.0)	0 (0.0)		
In all sexual relations with fixed partner***				0.250	0.251
No	9 (33.3)	18 (66.7)	0 (0.0)		
Yes	8 (47.1)	8 (47.1)	1 (5.9)		
In relation with hookups/casual encounters in the last 12 months****					0.350
No	14 (73.7)	4 (21.1)	1 (5.3)		
Yes	17 (38.6)	26 (59.1)	1 (2.3)		
In all sexual relations with hookups/casual encounters*****				0.250	0.251
No	9 (33.3)	18 (66.7)	0 (0.0)		
Yes	8 (47.1)	8 (47.1)	1 (5.9)		
Sexual relation					
With known individuals on the internet				0.192	0.131
No	32 (57.1)	22 (39.3)	2 (3.6)		
Yes	63 (46.0)	72 (52.6)	2 (1.5)		
With hookups/casual encounters in the last 12 months				0.721	0.058
No	33 (50.8)	30 (46.2)	2 (3.1)		
Yes	62 (48.4)	64 (50.0)	2 (1.6)		
Under the influence of alcohol				0.648	0.067
No	31 (54.4)	25 (43.9)	1 (1.8)		
Yes	64 (47.1)	69 (50.7)	3 (2.2)		
Under the influence of Marijuana				0.490	0.086
No	73 (49.7)	70 (47.6)	4 (2.7)		
Yes	22 (47.8)	24 (52.2)	0 (0.0)		
Under the influence of crack				0.979	0.015
No	94 (49.2)	93 (48.7)	4 (2.1)		
Yes	1 (50.0)	1 (50.0)	0 (0.0)		
Under the influence of cigarettes				0.461	0.090
No	79 (50.3)	74 (47.1)	4 (2.5)		
Yes	16 (44.4)	20 (55.6)	0 (0.0)		
Under the influence of cocaine				0.979	0.015
No	94 (49.2)	93 (48.7)	4 (2.1)		
Yes	1 (50.0)	1 (50.0)	0 (0.0)		

n, frequency of categories in absolute number; %, frequency of categories in percentage; ^€^The minimum wage in Brazil is approximately $291.12; ^£^Levels of knowledge: “good”, above 70% correct; “average”, 50–70% correct; and “poor”, below 50% correct; *Chi-square test or Fisher’s test; ^§^Cramer’s Correlation Coefficient; **Relative frequency calculated considering a sample of 125 students with a steady partner; ***Relative frequency calculated considering a sample of 46 students who used condoms during their last encounter with a steady partner in the past 12 months; ****Relative frequency calculated considering a sample of 68 students without a steady partner; *****Relative frequency calculated considering a sample of 44 students who used condoms in encounters with casual partners in the past 12 months.

## 4 Discussion

The present research revealed a high prevalence of inadequate knowledge about STIs and HIV/AIDS among the medical students participating in the study. This deficit is particularly concerning, considering the crucial role these future professionals will play in promoting health and preventing these diseases within the population.

The lack of knowledge about STIs and HIV/AIDS among medical students has also been demonstrated in other international studies (24–29). This knowledge gap is not only prevalent in developed countries but also in resource-limited settings, where there is often a lack of comprehensive sexual health education in medical curricula (25). The literature shows that inadequate and outdated information contributes to the perpetuation of myths and stigmas associated with STIs, which can result in sexual risk behaviors that compromise individual and collective health, as well as negatively impact the care of patients with HIV/AIDS and other STIs (25). Furthermore, it is necessary an integrated, multidisciplinary approach to sexual health education that spans the entire medical curriculum, ensuring that medical students not only acquire knowledge but also the skills to apply it in practice (24). This knowledge gap may reflect not only a lack of sexual education but also the absence of comprehensive curricular content. Moreover, it highlights the need for an integrated and multidisciplinary approach that transcends disciplines directly related to sexual health and permeates the entire medical curriculum (24).

Previous studies indicate that sociodemographic profiles can influence the adoption of risk behaviors, especially among young adults who are in a transitional phase characterized by greater independence and maturation. For instance, studies have shown that gender, sexual orientation, and cultural background can influence attitudes toward sexual health and the adoption of preventive behaviors (7, 22, 30, 31). Evidence suggests that among university students, women, despite having greater knowledge about STIs (7, 22), married individuals or those cohabiting with partners (32), people with lower socioeconomic status^32^, and those with higher levels of religiosity (30, 31) are more susceptible to sexual risk behaviors, such as inconsistent use of prevention methods, whether due to personal beliefs or inadequate sexual education. However, the present study did not demonstrate significant associations between the sociodemographic profile of participants and sexual risk behaviors, suggesting that other factors, such as social and academic contexts, may play a more relevant role in determining these practices. These results indicate the need to explore in future studies how different factors interact to influence sexual risk behavior among medical students.

When analyzing the sexual behavior of students, this study revealed that most exhibited some form of sexual risk behavior, particularly regarding inconsistent condom use in both stable and casual relationships. It was observed that condom use is considerably lower in stable relationships, corroborating the findings of other studies (32, 33). The inconsistency in condom use, particularly in stable relationships, is a significant risk factor since, even in monogamous relationships, there is always the possibility of exposure to STIs due to multiple factors, such as infidelity and lack of regular testing (34). In various countries, the adoption of sexual risk behaviors is often associated with negligence in adopting barrier methods in stable relationships, reflecting a false sense of security (35). These data suggest the need for interventions that promote awareness of the importance of consistent condom use, regardless of the type of relationship, as an essential part of a broader STI prevention strategy.

Another important point is that alcohol consumption (10) and the use of legal and illegal drugs (36), as well as the influence of the internet (37), have been identified as factors contributing to the adoption of sexual risk behaviors among youth. Studies indicate that alcohol consumption, which is more prevalent among men, is associated with the use of other psychoactive substances and engagement in sexual risk behaviors, such as lack of condom use and increased number of sexual partners. In this study, the majority of male individuals demonstrated sexual risk behavior and reported having sexual relations under the influence of alcohol, corroborating existing literature (10, 36). Additionally, the virtual environment and the use of dating apps have played an increasing role in facilitating sexual encounters (37), influencing the adoption of sexual risk behaviors and increasing exposure to STIs. The rise of online platforms for meeting potential sexual partners has been recognized as both a risk and an opportunity, as these platforms often promote risky sexual behaviors but can also be utilized for educational outreach (38).

Comparing the results of this study with research conducted in other countries, a similar trend in sexual risk behavior among medical students can be observed (26, 27, 29, 39). These findings reinforce the idea that, despite cultural and regional differences, medical students in various parts of the world are equally exposed to factors that make them vulnerable to risk behaviors, just like others in the same age group. This cross-cultural similarity suggests that medical curricula worldwide should address sexual health in a more comprehensive and context-specific manner (24). One of the main challenges for changing sexual behavior among medical students may be related to the dynamics of medical training itself. The intense workload, academic pressure, and lack of time to engage in educational activities can hinder awareness and the adoption of a healthy lifestyle and preventive practices. Studies have consistently shown that the demanding nature of medical education can lead to stress, burnout, and a reduction in the time available for engaging in health-promoting behaviors (40, 41). Furthermore, the institutional culture may not sufficiently value the importance of early sexual health education, contributing to students failing to recognize the seriousness of adopting risk behaviors. Therefore, it is essential for educational institutions to create spaces for debate and ongoing education on these topics, promoting a change in academic culture and reducing the vulnerability common in the studied age group. The implementation of pedagogical strategies that integrate interactive practices and real-life experiences, such as clinical simulations, debates, and workshops focused on awareness and prevention of STIs, may be useful in this process.

Such approaches can encourage students to reflect on their own sexual practices and reinforce preventive attitudes, as well as prepare them to interact with patients in a more empathetic and informed manner. This curricular reform, by combining theory and practice, has the potential to not only improve the knowledge of future doctors but also develop on psychoaffective skills, and in the long term, may contribute to reducing the rates of STI and HIV/AIDS transmission in society.

Finally, one of the main limitations of this study is its cross-sectional design, which prevents the establishing causal relationships between the analyzed factors and sexual risk behaviors. Furthermore, the use of self-administered questionnaires may have led to response bias, as participants may have underestimated their behaviors due to the sensitive nature of the topic. Another relevant limitation is that the sample consisted exclusively of medical students from a single institution, which may limit the generalization of results to other academic populations and regional contexts. Additionally, the data for this study were collected during the COVID-19 pandemic, a period marked by behavioral restrictions that could have influenced social interactions and sexual practices. Therefore, the results of this study should be interpreted in light of the context in which the data were collected. Future studies should consider more diverse samples and methodological approaches further explore the variables involved. Despite these limitations, this study contributes significantly to the field of medical education by identifying a knowledge gap regarding STIs and sexual risk behaviors among future physicians.

## 5 Conclusion

The results of this study highlight the high prevalence of sexual risk behaviors among medical students, especially regarding inconsistent condom use in both stable and casual relationships. Furthermore, a significant gap in knowledge about STIs and HIV/AIDS was identified; however, the level of knowledge does not appear to be the only determining factor in the adoption of these behaviors. These findings suggest the need to emphasize the importance of promoting a healthy lifestyle among students, including the early introduction of sexual education in the medical curriculum, aiming not only to improve the knowledge of future professionals but also to contribute to the reduction of STI and HIV/AIDS transmission in the general population, fostering more conscious and effective clinical practice.

## Data Availability

The raw data supporting the conclusions of this article will be made available by the authors upon reasonable request, without undue reservation.
